# Hepatic vulnerability is associated with post-hepatectomy liver failure and major morbidity after curative-intent resection of perihilar cholangiocarcinoma

**DOI:** 10.1186/s12893-026-04312-1

**Published:** 2026-11-03

**Authors:** Anna Mantas, Smiths S. Lueong, Sophia Mauerer, Antonia Krumm, Dieter P. Hoyer, Sophia M. Schmitz, Daniel Heise, Tom F. Ulmer, Jens Siveke, Ulf P. Neumann, Jan Bednarsch

**Affiliations:** 1 Department of Surgery and Transplantation, University Hospital Essen, Essen, Germany; 2 German Cancer Consortium (DKTK), Partner Site Essen/Düsseldorf, a partnership between German Cancer Research Center (DKFZ) and University Hospital Essen, Essen, Germany; 3 Bridge Institute of Experimental Tumor Therapy (BIT) and Division of Solid Tumor Translational Oncology (DKTK), West German Cancer Center, University Hospital Essen, University of Duisburg-Essen, Essen, Germany; 4 Department of Surgery, Maastricht University Medical Center (MUMC), Maastricht, Netherlands

**Keywords:** Hepatic Vulnerability, Perihilar Cholangiocarcinoma (pCCA), Post-hepatectomy liver failure

## Abstract

**Background:**

Post-hepatectomy liver failure (PHLF) remains a major determinant of morbidity after resection for perihilar cholangiocarcinoma (pCCA). We evaluated whether a composite HVS is associated with PHLF and major morbidity after resection for pCCA.

**Methods:**

Consecutive patients undergoing curative-intent resection for pCCA between 2010 and 2024 were retrospectively analyzed. The HVS combined preoperative hyperbilirubinemia, histological fibrosis and steatosis, with one point assigned to each component. The primary endpoint was PHLF according to ISGLS criteria; the secondary endpoint was major postoperative morbidity, defined as Clavien-Dindo grade ≥ III.

**Results:**

Among 203 patients, PHLF occurred in 59 (29.1%) and major complications in 105 patients (51.7%). PHLF increased stepwise with higher HVS category (21.0% vs. 33.8% vs. 42.4%, *p* = 0.033), with a significant trend across groups (*p* = 0.010). Major morbidity increased with higher score levels (44.0% vs. 57.7% vs. 63.6%, *p* = 0.071; trend *p* = 0.025). In multivariable analysis, HVS was independently associated with PHLF (OR 1.77, 95% CI 1.15–2.71, *p* = 0.009) and major morbidity (OR 1.51, 95% CI 0.99–2.27, *p* = 0.050). HVS showed a numerically higher AUC than its individual components in ROC analysis.

**Conclusions:**

A simple composite HVS combining biochemical and histological liver parameters is significantly associated with PHLF and major complications after pCCA resection. Further prospective and external validation is required to determine its potential clinical utility for risk stratification and patient selection in complex hepatobiliary surgery.

**Supplementary Information:**

The online version contains supplementary material available at https://doi.org/10.1186/s12893-026-04312-1.

## Introduction

Perihilar cholangiocarcinoma (pCCA) represents one of the most challenging entities in hepatobiliary surgery, requiring complex liver resections with substantial morbidity and mortality rates. Despite advances in surgical techniques and perioperative management, major complications and post-hepatectomy liver failure (PHLF) remain frequent and clinically relevant adverse events following curative-intent resection [1, 2].

PHLF is a multifactorial condition resulting from the interplay between patient-related, tumor-related and procedural factors. Among these, impaired liver regeneration has been identified as a central mechanism underlying postoperative liver dysfunction. In particular, pre-existing parenchymal liver alterations such as fibrosis and steatosis may compromise the regenerative capacity of the remnant liver and thereby increase the risk of PHLF and postoperative mortality [3, 4].

However, the clinical relevance of these histopathological alterations in resected pCCA patients remains largely undefined. To date, evidence specifically addressing the impact of liver parenchymal quality in pCCA is scarce with only very limited data available from dedicated cohort studies. While experimental and clinical data suggest that steatosis and fibrosis impair hepatic function and regeneration, available data in patients undergoing major liver resection for pCCA have not demonstrated a consistent association between these individual factors and postoperative outcomes [3]. This discrepancy highlights a fundamental limitation of current approaches, which typically evaluate isolated hepatic characteristics rather than the combined biological vulnerability of the liver.Given the multifactorial nature of liver dysfunction, it is plausible that the combined effect of several subclinical hepatic impairments rather than a single parameter determines postoperative risk. Therefore, a composite assessment of hepatic vulnerability integrating structural and functional aspects of the liver may better reflect the biological capacity for regeneration than individual variables alone.The aim of this study was to evaluate the impact of underlying hepatic vulnerability on postoperative outcomes after resection for pCCA. Specifically, we sought to assess the association of individual hepatic factors with PHLF and major morbidity and furthermore develop a composite hepatic vulnerability score (HVS) and investigate its independent association with postoperative outcomes in a well-characterized cohort of patients undergoing major liver resection for pCCA.

## Materials and methods

### Study design and patient population

This retrospective cohort study included all consecutive patients who underwent curative-intent resection for pCCA at the University Hospital Essen between January 2010 and December 2024. Patients were identified from a prospectively maintained institutional database. Only patients undergoing curative-intent surgery (R0/R1) and histologically confirmed pCCA were included. Patients with intrahepatic (iCCA) or distal cholangiocarcinoma and incomplete follow-up were excluded from analyses. Patients undergoing resection for recurrent pCCA disease were excluded to avoid confounding by prior treatments and altered liver parenchyma. The study was approved by the Ethics Committee of the Medical Faculty of the University of Duisburg-Essen, Germany, on 14 October 2024 (approval number: 24–12199-BO) and was conducted in accordance with the current version of the Declaration of Helsinki and the good clinical practice guidelines (ICH-GCP). Informed consent was waived due to the retrospective study design.

### Preoperative assessment

Preoperative staging was based on a structured work-up integrating clinical evaluation, laboratory testing and multiphasic cross-sectional imaging. Tumor burden, vascular involvement and the presence of metastatic disease were assessed using contrast-enhanced computed tomography (CT) and/or magnetic resonance imaging (MRI). For assessing a more detailed delineation of biliary anatomy and longitudinal ductal tumor extension, endoscopic retrograde cholangiopancreatography (ERCP) or magnetic resonance cholangiopancreatography (MRCP) was utilized. Resectability was determined on the basis of imaging-defined local tumor extent, future liver remnant (FLR) considerations and overall oncologic operability. All cases were reviewed by a multidisciplinary tumor conference. The final indication for surgery and the exact extent of resection was ultimately established by a senior hepatobiliary surgeon. Baseline operative risk and general physical status were documented according to the American Society of Anesthesiologists (ASA) classification.

### Data collection

Clinical, laboratory, operative and histopathological data were extracted from electronic medical records. Variables included demographic characteristics (age, sex, body mass index [BMI]), clinical variables (ASA classification, preoperative biliary drainage, cholangitis), laboratory parameters (hemoglobin, platelet count, INR, bilirubin, liver enzymes, C-reactive protein [CRP], CA19-9), tumor characteristics (grading, pT stage, nodal status, resection margin status) and operative variables (operative time and extent of resection). Biliary drainage was defined as any form of biliary decompression, including endoscopic stent placement, percutaneous transhepatic cholangiodrainage (PTCD) or metallic stent insertion. Histological assessment of the non-tumorous liver parenchyma, including fibrosis and steatosis, was performed on resection specimens by experienced hepatopathologists as part of routine histopathological evaluation. According to the institutional pathological grading system, steatosis was considered present when microvesicular and/or macrovesicular steatosis involved ≥ 5% of hepatocytes, corresponding to at least grade 1 steatosis.

### Definition of hepatic vulnerability score

The HVS was developed for this study as a simple composite score to reflect cumulative hepatic vulnerability. Hepatic vulnerability was defined as a composite score reflecting both functional and structural liver impairment. The score consists of three components: preoperative hyperbilirubinemia > 1.2 mg/dL, presence of histological fibrosis and presence of histological steatosis. Each component was assigned one point, resulting in a total score ranging from 0 to 3. Due to the low number of patients with a score of 3, the score was categorized into three groups (0, 1 and ≥ 2) for analysis. The HVS was analyzed both as an ordinal and as a dichotomized variable (score ≥ 2 vs. 0–1) to assess potential threshold effects. The definition of the HVS is provided in Supplementary Table 5.

### Outcome measures

The primary endpoint was PHLF, defined according to the criteria of the International Study Group of Liver Surgery (ISGLS). For analysis, PHLF was treated as a binary variable (presence vs. absence of PHLF). The secondary endpoint was major postoperative complications, defined as Clavien-Dindo grade ≥ III. Overall survival (OS) was defined as the time from surgery to death or last follow-up if the patient was alive. OS was recorded but not included in the primary analysis due to the focus on perioperative outcomes.

### Statistical analysis

Continuous variables are presented as median and interquartile range (IQR) and categorical variables are expressed as counts and percentages. Associations between categorical variables were assessed using chi-square tests or Fisher’s exact tests, as appropriate. Univariable logistic regression analysis was performed to evaluate associations between clinical variables and outcomes. Multivariable logistic regression models were constructed including statistically significant variables with *p* < 0.05 from univariate analysis to assess independent associations. Operative variables such as operative time and extent of resection were not included in the primary multivariable models, as they are considered procedural factors that may lie on the causal pathway and therefore represent mediating rather than confounding factors. Results are reported as odds ratios (ORs) with 95% confidence intervals (CIs). The hepatic vulnerability score was analyzed both as an ordinal variable and as a categorized variable. Receiver operating characteristic (ROC) analysis was performed and the area under the curve (AUC) was calculated to assess the discriminatory ability of the hepatic vulnerability score and its individual components for predicting PHLF. Cases with missing data were excluded from the respective analyses. Events per variable (EPV) were calculated for each multivariable logistic regression model. EPV was defined as the number of outcome events divided by the number of estimated model parameters in the respective complete-case model. Internal validation was performed using bootstrap resampling (1000 iterations) to assess model stability and optimism. All tests were two-sided, and a *p*-value < 0.05 was considered statistically significant. Statistical analyses were performed using SPSS software (SPSS Statistics version 24, IBM Corp., Armonk, NY, USA) and R software (R Foundation for Statistical Computing, Vienna, Austria).

## Results

### Patient cohort

A total of 203 patients undergoing curative-intent liver resection for pCCA were included in the analysis. The median age was 69 years (IQR 63–74) and 67% of patients were male. The vast majority of patients were classified as ASA II or III (95%). Preoperative biliary drainage was performed in 80.3% of patients and 29.1% presented with preoperative cholangitis. Histological assessment of the non-tumorous liver parenchyma revealed fibrosis in 48.2% and steatosis in 27.5% of evaluable cases. Tumor characteristics showed a predominance of advanced disease with 32.5% of patients presenting with Bismuth type IV tumors and 39.8% exhibiting nodal involvement. R1 resection was observed in 38.9% of cases. PHLF occurred in 29.1% of patients, including grade B/C in 18.2%. Major postoperative complications (Clavien-Dindo ≥ III) were observed in 51.7% of patients. The median OS was 19 months (95% CI 8–41). Detailed patient characteristics are summarized in Table 1.

**Table 1:** Patient characteristics and postoperative outcomes

**Variables**	**Derivation cohort**
Total patients	203
Demographics	
Sex, m/f (%)	136 (67)/67 (33)
Age (years)	69 (63–74)
BMI (kg/m2)	26 (23–28)
Preoperative systemic chemotherapy, n (%)	1 (0.5)
ASA, n (%)	
I	1 (0.5)
II	90 (44.3)
III	103 (50.7)
IV	9 (4.4)
V	0
Bismuth classification, n (%)	
I	31 (15.2)
II	18 (8.9)
IIIa	43 (21.1)
IIIb	45 (22.2)
IV	66 (32.5)
Preoperative biliary drainage, n (%)	163 (80.3)
Preoperative cholangitis, n (%)	59 (29.1)
Preoperative laboratory parameters	
Hemoglobin (g/dL)	12.8 (11.7–13.9)
Platelet count (/nL)	296 (236–374)
INR	0.98 (0.94–1.03)
AST (U/L)	50 (32–82)
ALT (U/L)	66 (42–122)
gGT (U/L)	428 (199–754)
Total bilirubin (mg/dL)	1.2 (0.6–2.8)
CRP (mg/dL)	1.4 (0.5–3.6)
CA19-9 (U/mL)	103 (33–361)
Histological characteristics	
Fibrosis, n (%)	82/170 (48.2)
Missing fibrosis information, n (%)	33 (16.3)
Steatosis, n (%)	47/171 (27.5)
Missing steatosis information, n (%)	32 (15.8)
Tumor grading, n(%)	
G1	11 (5.4)
G2	136 (67.3)
G3	55 (27.2)
G4	0
pT stage, n (%)	
1	25 (12.3)
2	102 (50.2)
3	11 (5.4)
4	65 (32)
Nodal involvement N1, n (%)	70 (39.8)
R1 resection, n (%)	79 (38.9)
Post-hepatectomy liver failure (ISGLS), n (%)	
No PHLF	144 (70.9)
Grade A	22 (10.8)
Grade B	16 (7.9)
Grade C	21 (10.3)
Postoperative complications (Clavien-Dindo), n (%)	
No complications	51 (25)
Grade I	16 (7.9)
Grade II	31 (15.3)
Grade IIIa + b	42 (20.7)
Grade IVa + b	27 (13.3)
Grade V	36 (17.7)
Median OS, months (95% CI)	19 (8–41)

Data presented as median and interquartile range if not noted otherwise

*ALT* alanine aminotransferase, *ASA* American society of anesthesiologists classification, *AST* aspartate aminotransferase, *BMI* body mass index, *CA19-9* carbohydrate antigen 19–9, *CI* confidence interval, *CRP* C-reactive protein, *gGT* gamma glutamyl transferase, *OS* overall survival, *INR* international normalized ratio

### Association of individual hepatic factors with outcomes

The presence of fibrosis and steatosis alone was not significantly associated with either PHLF or major postoperative complications. PHLF occurred in 37.8% of patients with fibrosis compared to 28.4% without fibrosis (*p* = 0.193), while major complications occurred in 62.2% vs. 52.3%, respectively (*p* = 0.192). The incidence of PHLF was higher in patients with steatosis (40.4% vs. 30.6%, *p* = 0.226), but these differences did not reach statistical significance. Major postoperative complications occurred more frequently in patients with steatosis (63.8% vs. 54.8%, *p* = 0.289) without statistical significance. In contrast, elevated preoperative bilirubin levels > 1.2 mg/dl were significantly associated with major postoperative complications (58.3% vs. 43.8%, *p* = 0.041) while no significant association with PHLF was observed. These results are summarized in Supplementary Table 1.

### Hepatic vulnerability score and postoperative outcomes

**Fig. 1:**
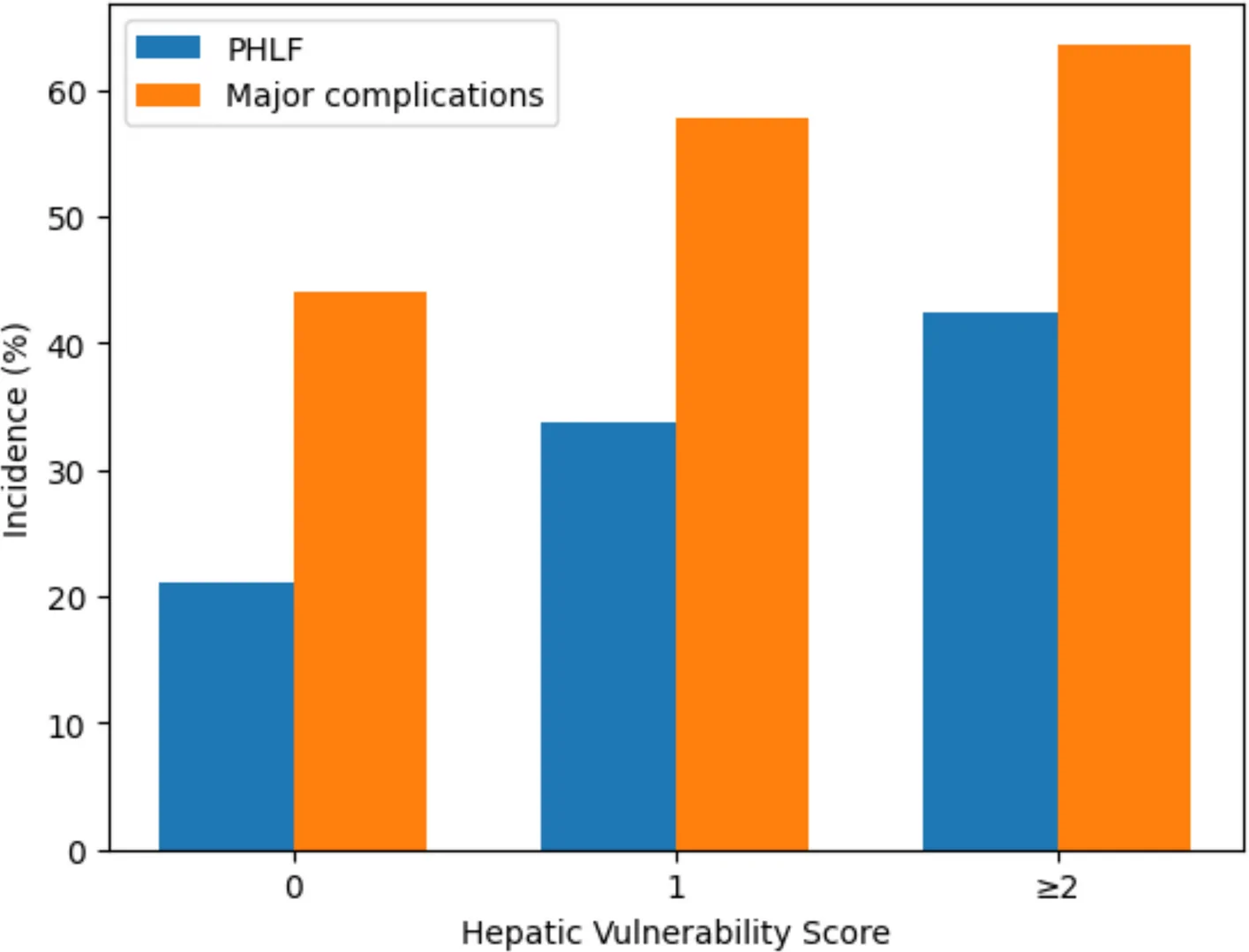
Incidence of PHLF and major postoperative complications according to hepatic vulnerability score. PHLF and major morbidity (Clavien-Dindo ≥ III) are displayed as grouped bars for each score category, demonstrating a stepwise increase with higher hepatic vulnerability. A significant trend was observed for both endpoints (p for trend: 0.01 for PHLF and 0.025 for major complications)

When combining individual hepatic factors into a composite HVS, a stepwise increase in postoperative risk was observed. The incidence of PHLF increased from 21% in patients with a score of 0 to 33.8% in patients with a score of 1 and 42.4% in patients with a score of ≥ 2 (*p* = 0.033). A significant linear trend was observed, indicating a progressive increase in PHLF with increasing hepatic vulnerability (p for trend = 0.010). A similar pattern was observed for major complications with rates increasing from 44% to 57.7% and 63.6%, respectively (*p* = 0.071; p for trend = 0.025, Fig. 1.)

### Predictors of PHLF

In univariable logistic regression analysis, female sex (OR 1.98, 95% CI 1.06–3.71, *p* = 0.033), low hemoglobin levels < 13 g/dl (OR 0.52, 95% CI 0.28–0.98, *p* = 0.042) and the hepatic vulnerability score (OR 1.69, 95% CI 1.12–2.54, *p* = 0.012) were significantly associated with an increased risk of PHLF. In multivariable analysis, the hepatic vulnerability score remained independently associated with PHLF (OR 1.77, 95% CI 1.15–2.71, *p* = 0.009) alongside low hemoglobin levels (OR 0.49, 95% CI 0.25–0.94, *p* = 0.033). Detailed results are shown in Table 2. Due to missing data in CA19-9 variables in 36 cases, this variable was excluded from logistic regression analysis. A sensitivity analysis was performed using reduced multivariable logistic regression models in which the hepatic vulnerability score was retained, and either sex or hemoglobin level was omitted (Supplementary Table 2). EPV was 19.7 for predicting PHLF (Supplementary Table 3).

**Table 2:** Logistic regression for PHLF

	**Univariate analysis**		**Multivariable analysis**	
	**OR (95% CI)**	***p*-value**	**OR (95% CI)**	***p*-value**
Demographics				
Sex (male = 1)	**1.98 (1.06–3.71)**	**0.033**	1.62 (0.84–3.12)	0.153
Age (≤ 65 years = 1)	0.60 (0.32–1.12)	0.108		
BMI (< 25 kg/m^2^ = 1)	0.66 (0.36–1.21)	0.180		
ASA (I/II = 1)	1.55 (0.83–2.88)	0.168		
Bismuth classification (I/II = 1)	2.14 (0.96–4.75)	0.062		
Preoperative biliary drainage (no = 1)	0.95 (0.44–2.01)	0.884		
Preoperative cholangitis (no = 1)	0.58 (0.31–1.11)	0.100		
Laboratory parameters				
Hemoglobin (< 13 g/dL = 1)	**0.52 (0.28–0.98)**	**0.042**	**0.49 (0.25–0.94)**	**0.033**
Platelet count (< 300/nL = 1)	1.68 (0.91–3.10)	0.096		
INR (< 1 = 1)	0.79 (0.43–1.46)	0.447		
AST (≤ 40 U/L = 1)	1.22 (0.65–2.29)	0.535		
ALT (≤ 40 U/L = 1)	1.69 (0.78–3.68)	0.183		
gGT (≤ 150 U/L = 1)	1.52 (0.68–3.44)	0.310		
CRP (< 1 mg/dL = 1)	1.81 (0.90–3.63)	0.097		
Histological characteristics				
Hepatic vulnerability score (per category)	**1.69 (1.12–2.54)**	**0.012**	**1.77 (1.15–2.71)**	**0.009**
Tumor grading (G1/G2 = 1)	1.45 (0.75–2.83)	0.272		
pT stage (T1/T2 = 1)	0.99 (0.74–1.31)	0.917		
Nodal involvement (no = 1)	1.41 (0.72–2.76)	0.315		
R1 resection (no = 1)	1.43 (0.74–2.76)	0.290		

Various parameters are associated with PHLF. Bold values indicate statistical significance (*p* < 0.05). CA19-9 was excluded from the analysis as it was missing in 36 cases

*ALT* alanine aminotransferase, *ASA* American society of anesthesiologists classification, *AST* aspartate aminotransferase, *BMI* body mass index, *CA19-9* carbohydrate antigen 19–9, *CRP* C-reactive protein, *gGT* gamma glutamyl transferase, *INR* international normalized ratio

### Predictors of major postoperative complications

Univariable analysis identified ASA III/IV classification (OR 2.67, 95% CI 1.51–4.72, *p* < 0.001), advanced Bismuth type III/IV (OR 2.51, 95% CI 1.29–4.90, *p* = 0.007), low hemoglobin level < 13 g/dl (OR 0.57, 95% CI 0.32–0.99, *p* = 0.045) and the hepatic vulnerability score (OR 1.57, 95% CI 1.07–2.30, *p* = 0.022) as significant variables for major complications. In multivariable analysis, high ASA classification (OR 2.65, 95% CI 1.45–4.83, *p* = 0.001), Bismuth classification (OR 2.50, 95% CI 1.22–5.12, *p* = 0.012) and the hepatic vulnerability score (OR 1.51, 95% CI 0.99–2.27, *p* = 0.050) remained independently associated with major postoperative complications. Results are shown in Table 3. Sensitivity analysis using reduced multivariable models showed broadly comparable effect estimates for the hepatic vulnerability score (Supplementary Table 4). EPV for predicting major complications was 35 (Supplementary Table 3).

**Table 3:** Logistic regression for major complications

	**Univariate analysis**		**Multivariable analysis**	
	**OR (95% CI)**	***p*-value**	**OR (95% CI)**	***p*-value**
Demographics				
Sex (male = 1)	1.48 (0.82–2.67)	0.195		
Age (≤ 65 years = 1)	1.38 (0.77–2.48)	0.275		
BMI (< 25 kg/m^2^ = 1)	1.17 (0.67–2.05)	0.576		
ASA (I/II = 1)	**2.67 (1.51–4.72)**	**< 0.001**	**2.65 (1.45–4.83)**	**0.001**
Bismuth classification (I/II = 1)	**2.51 (1.29–4.90)**	**0.007**	**2.50 (1.22–5.12)**	**0.012**
Preoperative biliary drainage (no = 1)	1.40 (0.70–2.80)	0.343		
Preoperative cholangitis (no = 1)	0.79 (0.43–1.45)	0.443		
Laboratory parameters				
Hemoglobin (< 13 g/dL = 1)	**0.57 (0.32–0.99)**	**0.045**	0.60 (0.33–1.10)	0.098
Platelet count (< 300/nL = 1)	1.16 (0.67–2.01)	0.600		
INR (< 1 = 1)	0.67 (0.38–1.16)	0.153		
AST (≤ 40 U/L = 1)	1.27 (0.72–2.23)	0.410		
ALT (≤ 40 U/L = 1)	0.93 (0.48–1.78)	0.818		
gGT (≤ 150 U/L = 1)	0.85 (0.42–1.70)	0.644		
CRP (< 1 mg/dL = 1)	1.50 (0.81–2.77)	0.196		
CA19-9 (≤ 100 U/mL = 1)	1.74 (0.94–3.22)	0.076		
Histological characteristics				
Hepatic vulnerability score (per category)	**1.57 (1.07–2.30)**	**0.022**	**1.51 (1.00–2.27)**	**0.050**
Tumor grading (G1/G2 = 1)	0.66 (0.35–1.24)	0.197		
pT stage (T1/T2 = 1)	1.26 (0.71–2.22)	0.435		
Nodal involvement (no = 1)	1.28 (0.70–2.35)	0.423		
R1 resection (no = 1)	1.41 (0.78–2.54)	0.260		

Various parameters are associated with major postoperative complications. Bold values indicate statistical significance (*p* < 0.05).

*ALT* alanine aminotransferase, *ASA* American society of anesthesiologists classification, *AST* aspartate aminotransferase, *BMI* body mass index, *CA19-9* carbohydrate antigen 19–9, *CRP* C-reactive protein, *gGT* gamma glutamyl transferase, *INR* international normalized ratio

### Discriminatory performance of hepatic vulnerability score

**Fig. 2:**
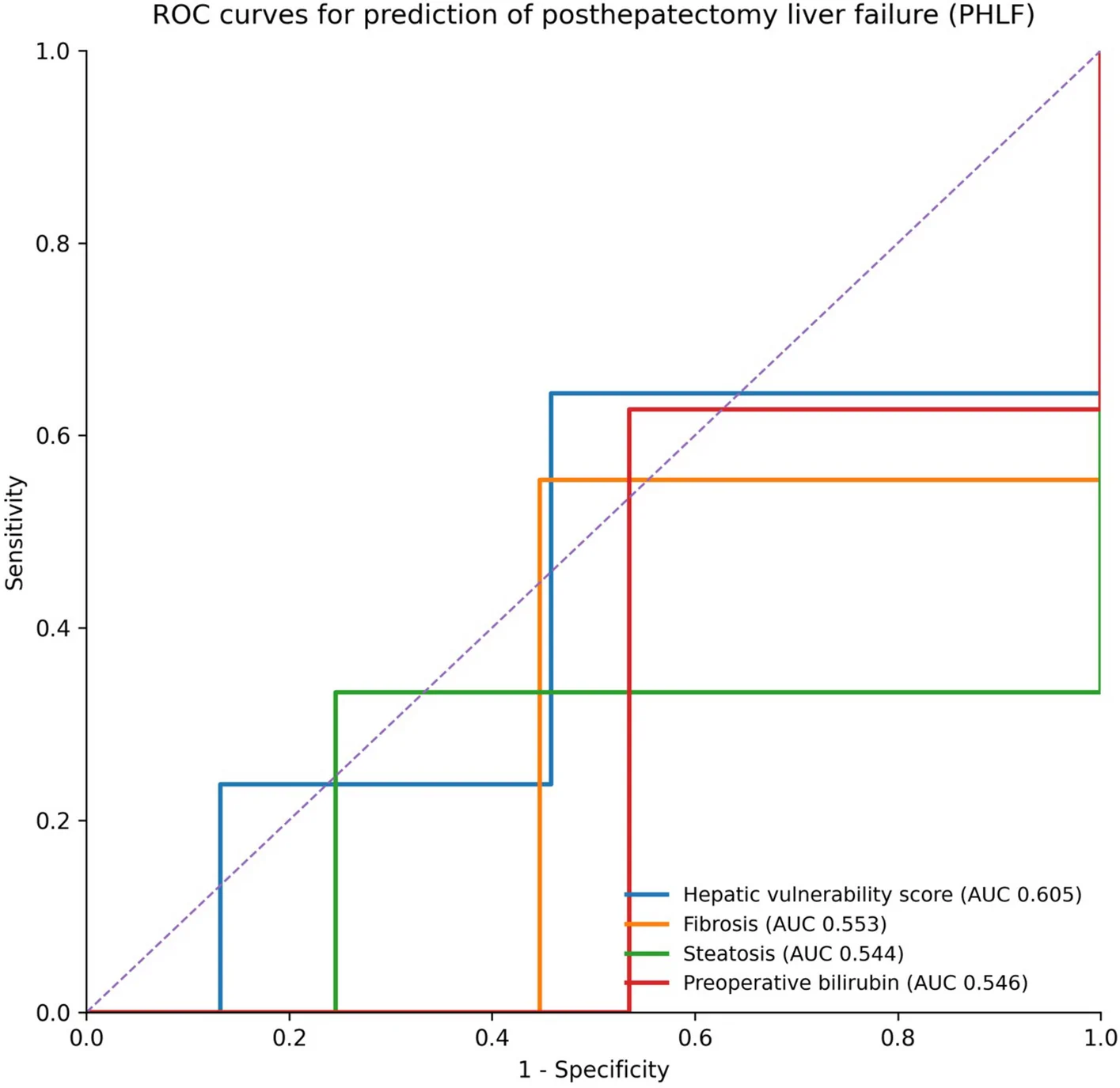
Receiver operating characteristic analysis for prediction of PHLF. ROC analysis demonstrated a modest discriminatory ability of the hepatic vulnerability score for prediction of PHLF with an AUC of 0.61. The composite score showed numerically higher discriminatory performance than its individual components, including fibrosis, steatosis and preoperative hyperbilirubinemia, which showed lower discriminatory performance with AUC values of 0.55, 0.54 and 0.55, respectively

ROC analysis demonstrated a modest ability of the hepatic vulnerability score to discriminate patients with and without PHLF (AUC 0.61). The HVS showed a numerically higher AUC (0.61) than its individual components, including fibrosis (AUC 0.55), steatosis (AUC 0.54) and preoperative bilirubin levels (AUC 0.55; Fig. 2).

### Internal Model validation

Bootstrap validation demonstrated minimal optimism (Delta Dxy = − 0.002) for PHLF, indicating stable model performance. The optimism-corrected discrimination remained modest, corresponding to an AUC of approximately 0.60. For major postoperative complications, bootstrap validation demonstrated modest model performance with an optimism-corrected AUC of approximately 0.59. While overall optimism was low, calibration estimates showed considerable variability, indicating limited model stability for this endpoint (Supplementary Figs. 1 and 2).

## Discussion

In this study, we demonstrate in a large European cohort of 203 patients that a composite hepatic vulnerability score, integrating preoperative hyperbilirubinemia, histological fibrosis and steatosis, is significantly associated with PHLF and major postoperative morbidity following curative-intent resection for pCCA. PHLF occurred in 29.1% and major postoperative complications in 51.7% of patients. These rates underline that postoperative liver-related morbidity remains a major clinical problem after pCCA resection and are in line with previous reports from van Keulen et al. identifying PHLF and severe postoperative morbidity as key determinants of adverse postoperative outcome in this setting [3].

Fibrosis occurs in 48.2% of all cases and steatosis was present in 27.5% of all patients, indicating that parenchymal liver alterations are common in pCCA patients. The individual components of the score showed limited and inconsistent associations with postoperative outcomes. Fibrosis alone was not significantly associated with PHLF or major complications, steatosis alone was also not significantly associated with either endpoint and elevated preoperative bilirubin was associated with major complications but not with PHLF. Our findings support previous observations that isolated hepatic parameters often have inconsistent predictive value in pCCA and related hepatobiliary settings and suggest that this inconsistency may be explained by the multifactorial nature of hepatic vulnerability, which is insufficiently captured by isolated parameters.

Hepatic steatosis is highly prevalent in the general population, affecting up to 30% of adults in Western countries with a rising incidence [5]. Despite the availability of various non-invasive modalities such as CT, MRI and ultrasonography, histopathological evaluation remains the gold standard for the detection and grading of hepatic steatosis [6, 7]. The use of resection specimen histology in the present study therefore provides a highly accurate assessment of liver parenchymal quality, representing a key methodological strength despite its limited preoperative availability. However, data on histologically assessed parenchymal liver alterations such as fibrosis or steatosis in specifically pCCA patients remain scarce. In a recent study from Kinzler et al., liver fibrosis was assessed in patients with iCCA, demonstrating high prevalences with up to 69% [8]. Chronic cholestasis may contribute to hepatic fibrogenesis through several complementary mechanisms. Impaired bile flow leads to intrahepatic accumulation of bile acids, which can induce hepatocellular and cholangiocellular injury, oxidative stress, mitochondrial dysfunction and inflammatory signaling. Experimental bile duct ligation models have shown that mechanical cholestasis induces biliary proliferation, myofibroblast activation and progressive periportal fibrosis [9–11]. This provides a biological rationale for integrating hyperbilirubinemia and histological liver alterations into a composite HVS.

PHLF is not only a frequent complication but also remains the most critical determinant of postoperative morbidity and mortality rates of up to 30% in patients undergoing resection for pCCA [12, 13]. Major postoperative complications remain frequent after extended resection for pCCA and are reported in up to 40–60% of patients in high-volume centers [2]. These complications are closely linked to postoperative liver dysfunction and contribute substantially to prolonged hospital stay, resource utilization and mortality [14].

Despite extensive research, reliable preoperative predictors of the above-mentioned outcomes on curative resected pCCA patients remain limited and commonly used parameters such as bilirubin levels or histological liver alterations have shown inconsistent predictive value across studies and on different hepatic malignancies [3, 15]. The key finding of this study is the stepwise increase in PHLF and major morbidity with increasing HVS. PHLF increased from 21% in patients with a score of 0 to 33.8% in those with a score of 1 and 42.4% in those with a score of ≥ 2 (*p* = 0.033, p for trend = 0.010), while major complications increased from 44% to 57.7% and 63.6%, respectively (*p* = 0.071, p for trend = 0.025). Patients with higher scores demonstrated substantially higher rates of PHLF, which was confirmed by logistic regression analysis showing a significant increase in risk per point increment of the score (OR 1.69, 95% CI 1.12–2.54, *p* = 0.012). This association was observed for both the primary and the secondary endpoint in multivariable analysis (PHLF: OR 1.77, 95% CI 1.15–2.71, *p* = 0.009; major complications: OR 1.51, 95% CI 0.99–2.27, *p* = 0.050), although the association with major complications was of borderline significance. Although the discriminative ability of the score was modest (AUC 0.61), it showed numerically higher discrimination for PHLF than its individual components, which is consistent with the concept that PHLF may result from the cumulative impact of functional and structural liver impairment rather than from single parameters alone.

From a pathophysiological perspective, this is plausible. Hyperbilirubinemia reflects impaired hepatocellular function and cholestasis, while fibrosis and steatosis represent structural alterations that reduce regenerative capacity and increase susceptibility to surgical stress. The combination of these factors likely creates a vulnerable hepatic microenvironment that predisposes to postoperative liver dysfunction. Therefore, assessing these factors in isolation may underestimate the true risk, whereas their integration provides a more comprehensive representation of hepatic reserve. Previous studies have identified several preoperative factors associated with resectability and postoperative risk, including advanced age, portal vein embolization, arterial infiltration exceeding 180° and elevated Carbohydrate antigen 19–9 (CA19-9), underscoring that risk assessment in pCCA is driven by multiple competing domains that are not captured by histological liver injury alone [16]. In addition, functional and volumetric parameters such as ICG clearance and future liver remnant volume have been associated with PHLF after resection for pCCA [17]. However, these factors do not directly capture the intrinsic biological quality of the liver parenchyma, which may represent an independent determinant of postoperative resilience. The HVS may therefore complement established clinical, tumor-related and technical risk factors by adding a liver-specific dimension to preoperative assessment. Future studies should investigate whether preoperatively available measures of hepatic vulnerability provide complementary information when integrated with dynamic liver function testing and FLR assessment. Importantly, fibrosis and steatosis were assessed histologically in the resection specimens and were therefore not routinely available preoperatively. Accordingly, the HVS in its present form should not be considered as a validated preoperative prediction tool or a direct measure of hepatic functional reserve. Although histopathological assessment remains the reference standard for defining parenchymal steatosis and fibrosis in the present study, the preoperative applicability of this concept could be enhanced by integrating non-invasive tools into the diagnostic work-up, such as conventional or quantitative ultrasonography for hepatic steatosis and transient elastography for fibrosis assessment, both of which have shown clinically relevant diagnostic or prognostic performance in a cohort of patients with HBV-related HCC [18]. If the histological components are obtained preoperatively, for example by liver biopsy, patients with high vulnerability scores may benefit from intensified preoperative optimization, more conservative resection strategies, or closer postoperative monitoring.

This concept is further supported by recent data from our work group demonstrating that body composition is associated with severe postoperative complications after curative resection for pCCA, highlighting that postoperative morbidity is influenced by multiple patient-related dimensions in addition to liver-specific factors [19]. Thus, hepatic vulnerability should not be interpreted as a stand-alone predictor, but rather as one complementary component within a broader multidimensional risk assessment framework.

From a practical perspective, the score may be useful in patients with borderline resectability or limited functional reserve. While hyperbilirubinemia is potentially modifiable through optimized biliary drainage, fibrosis and steatosis reflect more fixed structural alterations of the liver parenchyma. As staging laparoscopy can be performed in patients with pCCA, biopsy of the future liver remnant could be integrated into this procedure to obtain histological information before resection, thereby allowing preoperative calculation of the HVS.Patients with histological evidence of fibrosis or steatosis may then require particularly careful optimization of biliary drainage, conservative surgical planning and intensified postoperative monitoring.

The feasibility, risks and predictive value of such a biopsy-based approach should be evaluated in prospective studies.

However, several limitations of this study should be acknowledged. First, the retrospective design introduces the potential for selection bias. Second, fibrosis and steatosis were assessed histologically in the resection specimens and were therefore not available for preoperative calculation of the HVS in its present form. In addition, histological data were not available in all patients, which may have influenced the composition of the score. Furthermore, preoperative bilirubin levels in patients with pCCA may be influenced by tumor-related biliary obstruction and the timing and effectiveness of biliary drainage. Therefore, hyperbilirubinemia cannot be interpreted solely as a marker of intrinsic hepatic dysfunction and residual confounding by cholestasis and biliary drainage cannot be excluded. Third, the score assigns equal weight to all components. This approach was intentionally chosen to preserve the simplicity of the score and to avoid data-driven weighting within the same retrospective cohort. Nevertheless, equal weighting does not imply equivalent biological or prognostic importance of the individual components, and future validation studies should investigate whether differential weighting improves discriminatory performance. Furthermore, the discriminative performance of the score was moderate, indicating that additional factors likely contribute to postoperative outcomes. Additionally, systemic preoperative functional liver assessment using LiMAx testing, hepatobiliary scintigraphy or indocyanine green clearance (ICG) was not available and intraoperative ICG-based assessment was not routinely performed. Therefore, the hepatic vulnerability score could not be directly compared with established dynamic or imaging-based measures of liver function and future liver remnant capacity. This should be considered when interpreting the moderate discriminatory performance of the score as postoperative outcomes after major pCCA resection are likely influenced by additional functional, volumetric, technical and patient-related factors. Finally, external validation in independent cohorts is required before widespread clinical implementation.

Despite these limitations, this study provides a novel and clinically relevant perspective on risk assessment in perihilar cholangiocarcinoma. Our findings suggest that cumulative hepatic vulnerability, rather than isolated parenchymal alterations, is associated with PHLF after resection for pCCA.

## Supplementary Information

Supplementary Material 1.

## Data Availability

All relevant data were reported within the manuscript. The datasets generated and/or analyzed during the current study are available from the corresponding author on reasonable request. Dr. Bednarsch and Dr. Mantas had full access to all of the data in the study and take responsibility for the integrity of the data and the accuracy of the data analysis.

## Abbreviations

ALPPS: Associating liver partition and portal vein ligation for staged hepatectomy
ALT: Alanine aminotransferase
ASA: American society of anesthesiologists
AST: Aspartate aminotransferase
AUC: Area under the curve
BMI: Body mass index
CA19-9: Carbohydrate antigen 19–9
CEA: Carcinoembryonic antigen
CI: Confidence interval
CRLM: Colorectal liver metastases
CRP: C-reactive protein
CT: Computed tomography
EPV: Events per variable
ERCP: Endoscopic retrograde cholangiopancreatography
FFP: Fresh frozen plasma
FLR: Future liver remnant
GGT: Gamma glutamyl transferase
HCC: Hepatocellular carcinoma
HR: Hazard ratio
HVS: Hepatic vulnerability score
iCCA: Intrahepatic cholangiocarcinoma
IQR: Interquartile range
ISGLS: International Study Group on Liver Surgery
INR: International normalized ratio
MRCP: Magnetic resonance cholangiopancreatography
MRI: Magnetic resonance imaging
OR: Odds ratio
OS: Overall survival
pCCA: Perihilar cholangiocarcinoma
PHLF: Post-hepatectomy liver failure
PTCD: Percutaneous transhepatic cholangiodrainage
RFS: Recurrence-free survival
ROC: Receiver operating characteristic curve
