# Supplementary material for: Hepatic vulnerability is associated with post-hepatectomy liver failure and major morbidity after curative-intent resection of perihilar cholangiocarcinoma
Source: BMC Surg. 2026 Nov 3;26:743. doi: 10.1186/s12893-026-04312-1 (PMC13729593; doi:10.1186/s12893-026-04312-1)
Supplement: Supplementary file 1 [file 12893_2026_4312_MOESM1_ESM.docx]

**SUPPLEMENTARY MATERIAL**

**Supplementary Figure 1: Bootstrap-corrected calibration plot for prediction of PHLF.**

Calibration of the multivariable logistic regression model for prediction of PHLF was assessed using internal validation with 1,000 bootstrap resamples. The plot shows the agreement between predicted and observed probabilities of PHLF. The bias-corrected calibration curve demonstrated acceptable agreement with the ideal reference line, with a mean absolute error of 0.026.

**Supplementary Figure 2: Bootstrap-corrected calibration plot for prediction of major postoperative complications.**

Calibration of the multivariable logistic regression model for prediction of major postoperative complications was assessed using internal validation with 1,000 bootstrap resamples. The plot shows the agreement between predicted and observed probabilities of major complications. The bias-corrected calibration curve demonstrated moderate agreement with the ideal reference line, with a mean absolute error of 0.030.

**Supplementary Table 1: Association of individual hepatic factors with outcomes**

| **Variables** | **PHLF (%)** | **p-value** | **Major morbidity (%)** | **p-value** |
| --- | --- | --- | --- | --- |
| Fibrosis | 37.8 | 0.193 | 62.2 | 0.192 |
| No fibrosis | 28.4 |  | 52.3 |  |
| Steatosis | 40.4 | 0.226 | 63.8 | 0.289 |
| No steatosis | 30.6 |  | 54.8 |  |
| Elevated bilirubin | 32.2 | 0.244 | 58.3 | **0.041** |
| Normal bilirubin | 24.7 |  | 43.8 |  |

Data are presented as percentages. P-values were calculated using chi-square test or Fisher’s exact test, as appropriate. A p-value < 0.05 was considered statistically significant.

**Supplementary Table 2: Sensitivity analysis of the multivariable logistic regression model for prediction of PHLF**

| **Model** | **Included covariate** | **N** | **PHLF events** | **OR for HVS** | **95% CI** | **p-value** |
| --- | --- | --- | --- | --- | --- | --- |
| Primary multivariable model | Gender, hemoglobin, hepatic vulnerability score | 203 | 59 | 1.77 | 1.15-2.71 | 0.009 |
| Reduced model 1 | Gender, hepatic vulnerability score | 203 | 59 | 1.62 | 1.07-2.45 | 0.022 |
| Reduced model 2 | Hemoglobin, hepatic vulnerability score | 203 | 59 | 1.84 | 1.21-2.81 | 0.005 |

CA19-9 was excluded a priori due to missing data in 36 patients. ORs refer to the effect estimate of the hepatic vulnerability score. The HVS was retained in all models.

**Supplementary Table 3: Events per variable (EPV) for the multivariable regression models**

| **Model** | **Event definition** | **Number of events** | **Number of model parameters** | **EPV** |
| --- | --- | --- | --- | --- |
| Logistic regression for PHLF | PHLF (yes/no) | 59 | 3 | 19.7 |
| Logistic regression for major complications | Clavien-Dindo ≥III (yes/no) | 105 | 3 | 35 |

EPV, events per variable, was calculated as the number of outcome events divided by the number of estimated model parameters included in the multivariable logistic regression model.

**Supplementary Table 4: Sensitivity analysis of the multivariable logistic regression model for prediction of major complications**

| **Model** | **Included covariate** | **N** | **Major complication events** | **OR for HVS** | **95% CI** | **p-value** |
| --- | --- | --- | --- | --- | --- | --- |
| Primary multivariable model | ASA, Bismuth, hemoglobin, hepatic vulnerability score | 203 | 105 | 1.51 | 0.99-2.27 | 0.050 |
| Reduced model 1 | Bismuth, hemoglobin, hepatic vulnerability score | 203 | 105 | 1.61 | 1.08-2.41 | 0.020 |
| Reduced model 2 | ASA, hemoglobin, hepatic vulnerability score | 203 | 105 | 1.59 | 1.07-2.40 | 0.024 |
| Reduced model 3 | ASA, Bismuth, hepatic vulnerability score | 203 | 105 | 1.41 | 0.95-2.10 | 0.092 |

ORs refer to the effect estimate of the hepatic vulnerability score. The HVS was retained in all models.

**Supplementary Table 5: Definition of the Hepatic Vulnerability Score (HVS)**

| **Component** | **Definition** | **Points** |
| --- | --- | --- |
| Preoperative hyperbilirubinemia | Total bilirubin > 1.2 mg/dL before surgery | 1 |
| Hepatic fibrosis | Histopathological evidence of fibrosis in the resected liver specimen | 1 |
| Hepatic steatosis | Histopathological evidence of steatosis in the resected liver specimen | 1 |

**Score calculation:**

The HVS is calculated by summing the three components listed above.

**Possible score values:**

| **0** | **No vulnerability factors** |
| --- | --- |
| 1 | One vulnerability factor |
| 2 | Two vulnerability factors |
| 3 | Three vulnerability factors |
